# Accelerating research through reagent repositories: the genome editing example

**DOI:** 10.1186/s13059-015-0830-y

**Published:** 2015-11-20

**Authors:** J. Keith Joung, Daniel F. Voytas, Joanne Kamens

**Affiliations:** Molecular Pathology Unit, Center for Computational and Integrative Biology and Center for Cancer Research, Massachusetts General Hospital, Charlestown, MA 02129 USA; Department of Pathology, Harvard Medical School, Boston, MA 02115 USA; Department of Genetics, Cell Biology and Development, and Center for Genome Engineering, University of Minnesota, Minneapolis, MN 55455 USA; Addgene, Cambridge, MA 02139 USA

## Abstract

Keith Joung, Dan Voytas and Joanne Kamens share insights into how the genome editing field was advanced by early access to biological resources and the role in this process that plasmid repositories play.

## Changing science one plasmid at a time

The ability to build upon the findings of others is one of the cornerstones of science. We can, however, effectively use the achievements of our predecessors only if the data and the resources that they generated are easily accessible to us. In genomics, data deposition is almost a given, data formats and sharing frameworks are more and more standardized, and while data quality control is still necessary, it is not as time- and resource-consuming as similar processes for physical materials. Which is where public repositories of biological resources come in, to help the researchers cut through the red tape of material transfer agreements, and to ensure the quality of those resources. A number of such repositories exist to support molecular biology researchers, ranging from institutional, such as Harvard PlasmID Database or DNASU Plasmid Repository, through large state-funded ones, such as the National Institutes of Health’s PlasmID or Mammalian Gene Collection, to independent organizations, such as Addgene.

Addgene is a nonprofit plasmid repository that was founded in 2004 to overcome the obstacles with plasmid sharing. Shortly after its conception, Addgene launched a fruitful collaboration with the Zinc Finger Consortium, which was also in its infancy. Together, the two organizations have played an important role in the development of genome editing technologies. Here, we ask two founding members of the Zinc Finger Consortium, Keith Joung and Dan Voytas, and Addgene’s current Executive Director, Joanne Kamens, about this collaboration and its influence on the genome editing field.

### What inspired the foundation of Addgene? Why do we need this repository rather than researchers just requesting plasmids from other researchers?

**Joanne Kamens:** Addgene’s scientific founder, Melina Fan, was inspired by the almost universal experience of requesting published reagents and getting no response or the wrong plasmid. The waste of the vast resources stuck in lab freezers is what drives us every day. Addgene offers so many services and advantages over standard “walk down the hall” sharing. For starters, quality control and bar-coding of each plasmid ensures scientists get what they request. We are always curating with the help of our huge sharing community.

**Dan Voytas:** It is a considerable time and resource sink to send plasmids and reagents to many different investigators. The effort involves preparing and sending out materials as well as completing material transfer agreements. The burden becomes impossible when the reagents are highly sought after. There is no way we would have the resources to send out the 1500 or so TALEN kits that were requested from Addgene. No single lab could handle the tsunami of requests for CRISPR/Cas reagents.

### What impact has Addgene had on the genome editing field?

**Dan Voytas:** For many years, the field was frustrated by lack of efficient reagents to achieve targeted chromosome breaks. Efforts initially focused on zinc finger nucleases, then TALENs, then CRISPR/Cas. Each platform achieved new levels of efficacy. However, in order for the technology to be truly useful to the typical molecular biologist/geneticist, each new improvement had to be quickly disseminated. Addgene filled that need.

**Keith Joung:** Addgene played a very large role in enabling widespread distribution of genome editing reagents. When we started the Zinc Finger Consortium in 2005, a major goal of ours was to make the technology available to all academics interested in practicing it. Addgene enabled us to do this efficiently and effectively. I believe that the tone and practice we established early on in the field then led to others following suit as the TALEN and CRISPR/Cas9 technologies emerged.

### Are there any other fields where the repository had a big impact?

**Fig. 1:**
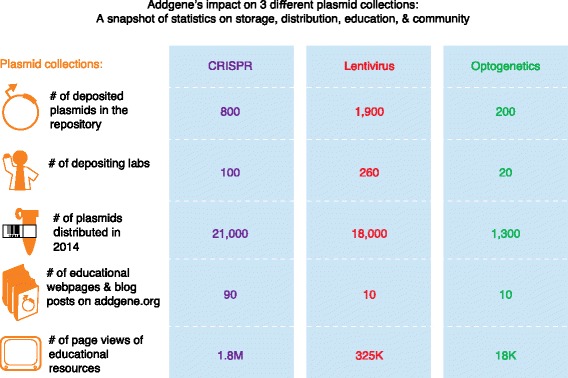
Examples of plasmid collections impacted by Addgene

**Joanne Kamens:** Addgene has impacted a wide variety of fields by not only helping to store and distribute plasmids, but also creating a community resource for protocols, educational materials, and science news. A few examples of plasmid collections and fields that Addgene has impacted are shown in Fig. 1. We recently updated our Viral Vector web pages to help scientists find commonly used envelope, packaging, and transfer plasmids, answer FAQs, and direct scientists to reliable protocols. Since we began creating our lentivirus resources, our web pages and related blog posts have been viewed over 325,000 times.

### What are some of the unique characteristics of the Addgene repository?

**Joanne Kamens:** Three of the most unique characteristics of Addgene are the diversity of the collection, the level of customer service, and its unique, nonprofit model. The collection’s diversity makes us a good place to start when looking for any plasmid. Researchers working in every organism and research field are depositing to Addgene. We recently opened a customer support office near London to enable us to more easily serve the international community of scientists. I say with some pride that Addgene’s customer service is unparalleled. We answer over 4500 emails and over 1000 phone calls each month. Most are logistics questions, but hundreds are technical service questions about plasmid selection or even “Why do my gel bands look like this?” Last, Addgene’s unique business model ensures that we are not dependent on grants or university support. This allows us to focus on service to the community. Also, as a nonprofit, we can make every decision based on fulfilling our mission: to accelerate research and discovery by improving access to useful research materials and information.

### Addgene’s resource has been steadily growing in the decade of its existence, but recent years have seen an especially large increase of submissions (from 25,000 in 2012, to 40,000 in 2015). Can you explain the reasons for this phenomenon?

**Fig. 2:**
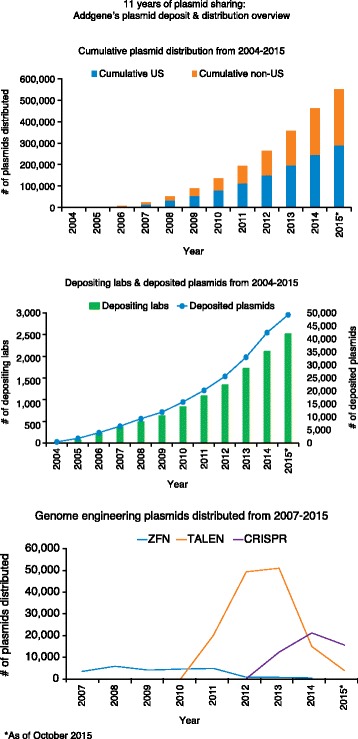
Growth of the Addgene resource

**Joanne Kamens:** In early 2013 Addgene hit a tipping point. That year Addgene was able to dramatically increase outreach activities (Fig. 2). Additionally, it was the year when most of the labs doing the early work on CRISPR/Cas9-mediated genome engineering committed to making their plasmids easily accessible via Addgene. Addgene’s increased visibility in the research community was driven by our own team raising awareness and by scientists finding Addgene because they were interested in the new genome engineering technologies. Kudos to the scientists who deposited with Addgene before publication so plasmid numbers could be included in those first CRISPR papers. Once scientists find us, our great plasmid and information resources keep them engaged.

### The Zinc Finger Consortium has been collaborating with Addgene almost from its very beginning. Can you tell us how this collaboration started?

**Dan Voytas:** Keith learned about my efforts (initially unsuccessful) to make ZFNs for the targeted modification of plant genomes. He suggested that we work together to come up with a reliable, easy-to-use platform to make effective ZFNs. I invited Keith to visit me at Iowa State University, and during that meeting we crafted the idea for establishing a Zinc Finger Consortium to achieve this end. We felt from the onset that the tools of genome engineering should be broadly available. Hence, we began a relationship with Addgene from the onset to make the reagents available.

**Keith Joung:** As Dan says, the Zinc Finger Consortium was born in October 2005 during a meeting he and I had at Iowa State University. We knew that we wanted to make the reagents we would be generating available to academics but I have to admit that we didn’t really know in the early days exactly how we would do it. Around the same time as we were beginning to plan for distributing our initial set of reagents, I was contacted by someone from Addgene who wanted to tell me about their services. I remember calling Dan after that meeting and saying to him that I thought we had an answer for how to distribute the reagents. We committed our initial set of plasmids to Addgene and then had a productive relationship going forward for all of our future reagents.

### What advice would you give to researchers who would like to start a similar type of collaboration with Addgene?

**Joanne Kamens:** We are extremely eager to collaborate with all kinds of labs, consortia, and communities to create special collections. The Zinc Finger Consortium really helped us to get the ball rolling and serves as a model for how Addgene can work with groups to promote their useful reagents. Call us anytime and we will assign an Addgene scientist to your project. We can’t possibly have in-house expertise in all the areas, so we really appreciate having thought leaders who are familiar with the science helping us bring new collections online. The Addgene Special Collections page gives a taste of the many different ways scientists have worked with us to deposit collections, encourage their colleagues to deposit, and to provide supporting information for the community (https://www.addgene.org/special-collections/).

### We expect to see a growing number of screening studies utilizing CRISPR/Cas technology. Given the numbers of sgRNAs used in a screen like this, do you expect a large increase of depositions to Addgene? Will it be scalable enough to handle those?

**Joanne Kamens:** Addgene has been actively preparing to take larger plasmid collections and screening libraries. We are currently optimizing the technology in house to amplify pooled libraries to ensure an endless supply of high-quality CRISPR libraries will be available to an increasing number of interested laboratories. Pooled libraries are distributing very well and more are being deposited. Last year, Dr. Michael Davidson from Florida State University contacted us. He wanted Addgene to take his entire 3000 plasmid fluorescent protein collection as a legacy. This was an order of magnitude bigger than any other deposit we had taken especially because the utility of this amazing collection meant that it needed to be able to be distributed individually, not just as a full collection. We learned a lot getting this collection online and are in the process of automating many of our processes to make sure we can accept collections of any size.

### Do you think similar repositories are needed for other types of materials? If so, what advice would you give to people wanting to start one?

**Dan Voytas:** It is unclear to me if multiple repositories are necessary. Technology changes quickly, and as long as one repository is able to adapt to meet the community’s needs, that should suffice.

**Keith Joung:** As genome editing nucleases continue to be widely adopted, it may become important to establish repositories for cells and organisms modified by these technologies. Although I suppose that it might also become so simple to generate these cells and organisms that doing so may be unnecessary as well. It will be exciting to see what happens moving forward.

**Joanne Kamens:** I went back to Addgene’s founders to get expert input on this one. Melina Fan says, “When starting a repository, trust is your most important asset. To build trust, you need to meticulously track your samples, conduct vigorous quality control, and provide excellent scientific support. To seed the repository, actively pursue the samples that you believe are important for the research community to share.” Benjie Chen adds, “Come up with a plan to be self-sustaining, make sure you are being realistic to yourself and the model is attainable. Then, commit to it and work on it, and be persistent even when times are tough.” Finally, Ken Fan offers this advice, “Bio-repositories can be expensive to launch and to operate. Before starting a new repository, make sure to evaluate the short-term and long-term potential for financial self-sustainability, including start-up expenses, expected revenue and operating costs. Also, given the challenges with building a new repository, make sure your mission and personal objectives are clear and focused.” I will add a piece of my own advice: hire great people who enjoy working together. Addgene’s most valuable assets are the talented Addgenies who work hard to serve the scientific community.

### What lies in the future for reagent repositories in general and Addgene in particular?

**Dan Voytas:** In the early days of the genome engineering revolution, we witnessed the development of tools/reagents that are broadly useful across diverse organisms. I anticipate that the next phase will involve the development and dissemination of reagents and vectors that achieve effective targeted modification in a subset of organisms (plants, insects, vertebrates). The demand for a given, specialized reagent will be less; however, access to these reagents will be very important for advancing science in many research communities.

**Keith Joung:** It is gratifying to see the model of sharing reagents so widely practiced now by the genome editing field. When Dan and I started doing this with the Zinc Finger Consortium almost a decade ago, I know we believed that it was important to do but I don’t think we quite appreciated how much of an impact our actions would have in setting the tone and approach for the larger field.

**Joanne Kamens:** We recently moved into a new, larger facility which will allow us to expand in new directions. Part of our new space is being outfitted as a BL2+ laboratory to enable mammalian cell work at Addgene for the first time. We will also be adding a new research team to pursue new projects that require more technical benchwork than we do now. Addgene provides an enormous amount of technical customer service. With this new lab space, our scientists will be able to test out protocols and reagents, allowing us to better support the community with protocol and reagent advice. Another project we are pursuing is being able to offer our plasmids in different formats. I hope to be able to tell you much more about this in 2016. We are incredibly excited to be in a position to consider new ways to help scientists accelerate their research.

